# Chinese herbal formulae for the treatment of menopausal hot flushes: A systematic review and meta-analysis

**DOI:** 10.1371/journal.pone.0222383

**Published:** 2019-09-19

**Authors:** Mingdi Li, Andrew Hung, George Binh Lenon, Angela Wei Hong Yang

**Affiliations:** 1 School of Health and Biomedical Sciences, RMIT University, Bundoora, Victoria, Australia; 2 School of Science, RMIT University, Melbourne, Victoria, Australia; Macau University of Science and Technology, MACAO

## Abstract

**Objectives:**

This systematic review aimed to evaluate the therapeutic effects and safety of Chinese herbal medicine (CHM) formulae for managing menopausal hot flushes (MHF).

**Methods:**

Seven English and Chinese databases were searched for studies from respective inceptions to February 2019. Randomized controlled trials investigating the clinical effects and safety of CHM formulae on MHF were considered for inclusion. The outcomes of subjective feelings (MHF and quality of life), objective changes (hormones and peripheral blood flow) and safety were analyzed. The most frequently prescribed formulae and herbs were summarized.

**Results:**

Nineteen randomized clinical trials involving 2469 patients were included. When compared to menopausal hormone therapy, CHM had similar effects to menopausal hormone therapy on total effectiveness rate (OR 1.41, 95% CI 0.84 to 2.35) and total Kupperman index (KI) score (SMD -0.13, 95% CI -0.61 to 0.36), and could significantly reduce vasomotor symptom score (MD -0.43, 95% CI -0.55 to -0.31) and upper-body peripheral blood flow (MD -3.56, 95% CI -5.14 to -1.98 under the jaw, MD -7.10, 95% CI -11.01 to -3.19 in the fingertip). When compared to placebo, CHM could reduce MHF severity (MD -0.70, 95% CI-1.00 to -0.40) and improve total KI score (MD -12.61, 95% CI -15.21 to -10.01). However, no statistically significant changes to hormone levels were detected. Most commonly seen adverse events were mild gastrointestinal tract reactions. The most popularly studied formula was Kun Tai capsule and the most frequently prescribed herb was Bai shao (Paeoniae Radix Alba, *Paeonia lactiflora* Pall.). More than 50% included studies had low risks of bias in the domains of selection, performance, attrition and reporting.

**Conclusions:**

This review indicated that CHM formulae were safe to be applied in MHF females and able to improve MHF-related symptom scores as well as the peripheral blood flow. Further studies should focus on specific formulae.

## Introduction

Menopausal hot flushes (MHF) refer to spontaneous but transient erythema and warm or burning sensation on the face and neck which affects 60–70% women during the menopausal period [1]. It has become the primary reason for females to seek medical help [2]. Despite the observation that symptoms will resolve spontaneously and halt ultimately even without any treatments, the average persistent duration of MHF is 2.5 years [3] and in approximately 10–15% cases it could last up to decades or even longer after menopause [4]. Therefore, MHF is commonly considered as a negative domain of the quality of life (QoL) in middle-aged women which can vitally impact on concentration and emotional abilities during daily activities [5]. Although the precise mechanism of MHF is not known, a sudden reduction in estrogen level, changes in neurotransmitters (serotonin and norepinephrine), calcitonin gene-related peptide (CGRP) and neurokinin-B are among those considered as players in the pathomechanism of MHF [6].

Current therapies including menopausal hormone therapy (or hormone replacement therapy, HRT) and paroxetine (selective serotonin reuptake inhibitors) are the recommended therapies for MHF by the North American Menopause Society (NAMS) [7, 8]. Although these therapies are efficacious, they are not without adverse events (AEs), such as nausea, dizziness and dry mouth. Additionally, HRT is contraindicated with hormone-dependent diseases (e.g. breast cancer) and past discontinuation of HRT may contribute to a rebound in hot flushes [9–11].

Therefore, a tremendous effort has been invested to search for natural products. The Study of Women’s Health Across the Nation (United States), a longitudinal six-year prospective cohort study, reported that approximately 80% of 3302 investigated females during menopausal transition have used complementary and alternative medicine (CAM) during the six-year investigation period [12]. Among these CAM, Chinese herbal medicine (CHM) is a popularly prescribed therapy for MHF management in real-life clinical practice and widely studied in clinical trials. However, to date, they have not been systematically reviewed. This study aimed to conduct a systematic review which offers a comprehensive evaluation of the therapeutic effects and safety of CHM for MHF and identify the most commonly used CHM formulae and individual herbs for MHF.

## Methods

This systematic review was conducted following the requirements of the Cochrane Handbook [13] and the PRISMA checklist (S1 PRISMA checklist) [14].

### Data sources and search strategies

Five English databases (Cochrane Library, PubMed, EMBASE, CINAHL and AMED) and two Chinese databases (CNKI and sinoMed) were searched for studies published in English and Chinese from their respective inceptions to May 2018, and updated in February 2019, by using MeSH terms of MHF and CHM and their synonyms as keywords. The results were exported into and managed by EndNote library. The reference lists of relevant trials and reviews were screened for additional studies. Sample search strategies are provided in S1 Table.

### Study screening and selection criteria

Articles retrieved for evaluation were assessed by two independent reviewers. One reviewer performed the screening of the title and abstract and two independent reviewers screened the full-text of potential records for eligibility. The disagreement was discussed between the two reviewers. If an agreement could not be reached between the two reviewers, the discrepancies were discussed with the third party. All the searched articles were screened and evaluated according to inclusion and exclusion criteria. The articles were considered for inclusion if they 1) were randomized controlled trials (RCTs), 2) recruited female participants who were experiencing MHF during or after natural or artificial menopause transition, 3) contained CHM formulae in any forms (decoction or capsule) as treatment, and 4) included conventional treatment (hormone or western medication), placebo or no treatment as a control intervention.

Articles with ineligible participant: 1) hot flushes related to breast cancer or drug-induced hot flushes or 2) no diagnostic criteria for MHF were excluded. Articles were excluded if they used any of following CHM interventions: 1) single herbs, chemical compounds extracted from herbs, and supplements; or 2) individualized CHM formulae for participants (i.e. modification of formula according to symptoms); or 3) external application of herbal formulae. Articles with control group other than placebo, conventional treatment (hormone therapy or western medication) or no treatment were not considered for inclusion in this review. Studies involving inappropriate comparison leading to the effects of CHM formulae could not be determined were also excluded. Co-intervention was not considered for inclusion in this review.

### Data extraction

Data were extracted from included studies by two independent reviewers. The data extracted to a predesigned data collection form included details about the study design, setting, sample size, participants’ age, diagnosis, interventions, duration and outcome measures.

In this review, the primary outcome measures were the subjective feelings of MHF including effectiveness rate, frequency, severity and score (i.e. an MHF score was calculated by multiplying the number of MHF severity score by the frequency of daily MHF) of MHF symptom, the Kupperman index (KI) score (limited to the total score and vasomotor domain score) and the QoL (limited to the total score and vasomotor domain score). The secondary outcome measures were the objective measurements of MHF including menopause-related hormone levels (i.e. follicle-stimulating hormone [FSH], luteinizing hormone [LH] and estradiol [E_2_]) and peripheral blood flow. Included studies containing these data were considered for further data analysis. The most commonly studied formulae, herb pairs and herbs in the included studies were descriptively summarized. The compatibility of herb pairs was networked using Gephi (https://gephi.org/).

### Data synthesis and analysis

The quality of included RCTs was assessed against the risk of bias (e.g. selection, performance, attrition, detection and reporting biases) according to the Cochrane Handbook [13] independently by two reviewers. The disagreement was discussed between the two reviewers or with a third party.

The extracted quantitative data from published studies were analyzed using RevMan 5.3 [15]. Dichotomous data (i.e. effectiveness rate) were weighted by odds ratio (OR). Continuous data of the hormone levels were weighted by the mean difference (MD) with 95% confidence intervals (CI). The standardized mean difference (SMD) was utilized instead of MD when different scales were used in more than one study for the same outcome measures including MHF relief (frequency, severity and score), KI score and QoL. Heterogeneity was assessed statistically using I^2^. To minimize the potential heterogeneity, random effects were applied where I^2^ was over 50% [13]. The descriptive synthesis was also used to aid in data presentation when appropriate. Sensitivity analysis was also considered where applicable. Rosenthal Failsafe-N were calculated using Meta-Essentials 1.4 version [16] for publication bias where more than one study was included in a outcome measure [17].

## Results

**Fig 1:**
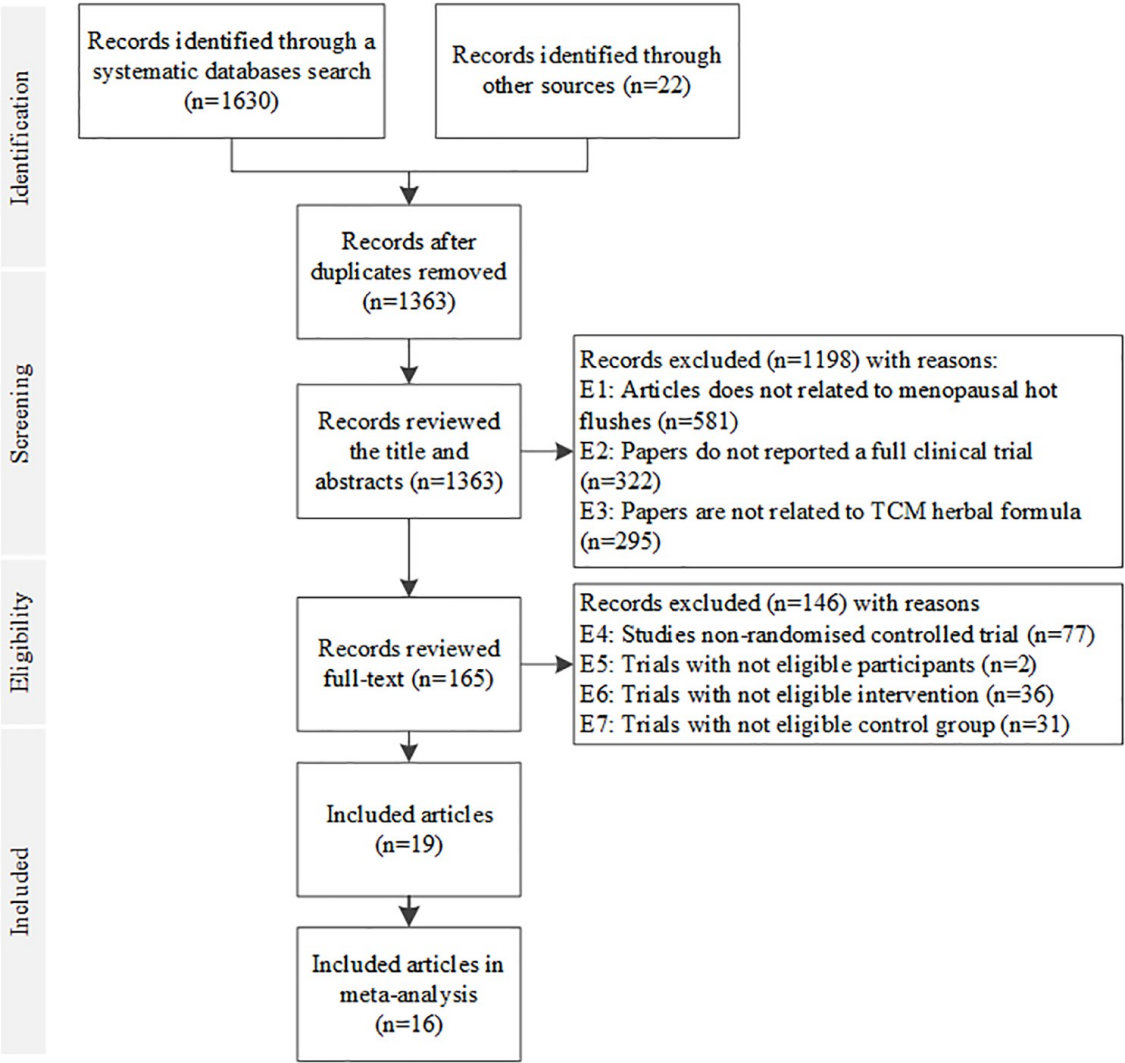
PRISMA flow chart on study selection procedures of Chinese herbal medicines for the management of menopausal hot flushes.

A total of 1630 papers were obtained from the database search and 22 articles were found through manual search. Nineteen articles met inclusion criteria and were included in this review [18–36] and 16 were included in the meta-analysis [18–26, 28, 30, 31, 33–36]. Three were not included in the meta analysis due to lack of data [27, 29, 32]. The selection process for included studies with exclusion details is shown in Fig 1.

### Description of studies

All of the 19 included studies are RCTs which recruited 2469 participants (1365 in the CHM groups, 1024 in the control groups, and one study did not specify the group allocation of 80 participants in the recruitment stage [23]) and analyzed 2309 participants at the end of treatment. These trials were conducted worldwide including 11 in mainland China [18–24, 26, 28, 34, 36], two in Hong Kong [30, 35], two in Australia [27, 33], two in America [29, 32], one in Japan [25] and one in Switzerland [31]. Thirteen of them were published in English [18, 24, 25, 27–36] and six published in Chinese [19–23, 26].

All the involved studies involved female participants only. They were diagnosed with MHF: 12 studies applied laboratory tests of hormone level to determine the menopause transition [18, 19, 21, 23–25, 27, 28, 30–33]; hot flushes were all diagnosed by participants’ subjective feeling. The duration of MHF intervention varied from 4 weeks to 12 months. Regarding the types of herbal medicine, seven RCTs used CHM in granule [25, 27–29, 34–36], five in capsule [19, 23, 24, 30, 31], three in decoction [20, 22, 26], two in tablet [32, 33] and two in honey pill form [18, 21]. The number of ingredients of CHM formulae ranged from 2 to 31 herbs. Among the 19 studies, nine of them compared CHM with HRT [18–26] and ten compared CHM with placebo [27–36]. No studies comparing CHM with no treatment were identified. The characteristics of included studies are summarized in Table 1 and details of ingredients of CHM formulae are provided in S2 Table.

**Table 1:** Characteristics of included studies of Chinese herbal formulae on menopausal hot flushes.

Study ID	Setting	Sample size/ EoT	Diagnosis of menopause transition	Diagnosis for MHF	Duration	Interventions		Outcome measures
						T	C	
CHM vs HRT								
**Azizi et al. 2011 [18]**	Hospital of university	48 (T: 24, C: 24)/37 (T_1_: 22, C: 15)	FSH 10 IU/L or menstrual irregularities	≥ 3 symptoms in KI index	2 months	Kun Bao Wan honey pill, 1 sachet, twice/day	Steady conjugated estrogen 0.625 mg/day + medroxy progesterone acetate 2 mg/day or conjugated estrogen 0.625 mg/day for 28 days + medroxy progesterone acetate (NS form), 4 mg/day in the last 12 days repeated after 7 days of rest	KI scale (overall), FSH, E_2_, number of symptoms before and after treatment, AE
**Chen 2014 [19]**	Hospital	100/100 (T: 50, C: 50)	Amenorrhea ≥ 3 months, FSH ≥ 20 IU/L, E_2_ ≤ 30 pg/mL	≥ 3 MHF per day	3 months	Kun Tai capsule, 4 capsules, 3 times/day	E_2_ valerate tablet, 0.5 mg, once/day	Modified KI scale, MHF effectiveness rate, hormone (E_2_), MI
**Fu et al. 2015a [20]**	Health centre	98/98 (T: 50, C: 48)	According to textbook ‘Obstetrics and Gynecology’ 7th edition	KI index	1 month	Chinese herbal formula, 100ml, twice/day	Nilestriol tablet, 2 mg/week	Overall effectiveness rate, modified KI scale, AE
**Li et al.** **2018 [21]**	Clinic of hospital	160/160 (T: 80, C: 80)	According to textbook ‘Obstetrics and Gynecology’ 7th edition, FSH ≥ 40 IU/L, E_2_ ≤ 30 pg/mL	Total KI index ≥ 10, one symptom score ≥ 2, symptomatic	3 months	Ding Kun Dan, 1 pill, twice/day	Tibolone tablet, 2.5 mg/day	Effectiveness rate (modified KI scale), prevalence of osteoporosis, MHF and sweating, hormone (FSH, LH, E_2_), liver and kidney function, AE
**Liu 2008 [22]**	Hospital	64/64 (T: 32, C: 32)	According to textbook ‘Obstetrics and Gynecology’ 5th edition	Symptomatic	4 weeks	Modified Dan Zhi Xiao Yao San, 1 bag, once/day	Premarin tablet (conjugated estrogens 0.3 mg, three times/day) for 28 days + medroxyprogesterone acetate (NS form) 10 mg/day for 5 days	Effectiveness rate of symptoms, AE
**Luan et al. 2004 [23]**	Hospital	80/66 (T: 33, C: 33)	Natural amenorrhea ≥ 3 months, FSH ≥ 40 mIU/mL, E_2_ ≤ 30 μg/L	≥ 3 MHF and sweating per day	3 months	Kun Tai capsule, N/A	E_2_ valerate tablet, 0.5 mg	Modified KI scale, hormone (FSH, E_2_), biochemical indicators, AE
**Sun et al. 2018 [24]**	Clinical centers	390 (T: 196, C: 194)/318 (T: 159, C: 159)	Amenorrhea for 3 months to 3 years or hysterectomy within 3 years, E_2_ < 30 ng/L, FSH > 40 IU/L	Symptomatic	12 months	He Yan Kun Tai Capsule, 4 capsules, twice/day	Conjugated estrogen tablets (average daily dose 0.45 mg, alternate 0.6 mg and 0.3 mg), for patients without hysterectomy + medroxyprogesterone acetate tablets (2 mg/day)	KI scale, hot flush score, insomnia score, MENQOL, AE
**Ushiroyama et al. 2005 [25]**	College	140 (T: 70, C: 70)/131 (T: 67, C: 64)	Amenorrhea ≥ 6 months, FSH > 30 mIU/mL, LH > 20 mIU/mL, E_2_ < 20 pg/mL	With climacteric complaints	1 month	Gui Zhi Fu Ling Wan granule, 7.5 g/day	Oral HRT consisting of combined conjugated equine estrogen (Premarin tablet, 0.625 mg/day) and medroxy progesterone acetate (Provera tablet, 2.5 mg/day)	Blood flow (under the jaw, fingertip, toe tip)
**Zhou & Li 2016 [26]**	Hospital	66/66 (T: 32, C: 34)	According to textbook ‘Obstetrics and Gynecology’ 7th edition	KI index	1 month	Chinese herbal formula, 100 mL, twice/day	E_2_ valerate tablet, 1 mg, once/day	Effectiveness rate, modified KI scale, effectiveness rate of symptom improvement (overall), AE
CHM vs placebo								
**Davis et al. 2001 [27]**	Medical center	78 (T: 42, C: 36)/55 (T: 28, C: 27)	Postmenopausal (>12 months amenorrhea, FSH > 25 IU/L)	≥ 14 MHF or night sweats per week	12 weeks	CHM formula granule, 1 sachet, twice/day	Placebo, 1 sachet, twice/day	Changing in frequency of hot flushes and night sweats (daily diary), MENQOL, total urinary diadzein and genistein excretion, AE
**Fu et al. 2015b [28]**	Hospital of university	389 (T_1.1_: 49, T_1.2_: 50, T_1.3_: 51 C_1_: 49; T_2.1_: 48, T_2.2_: 47, T_2.3_: 47, C_2_: 48)/383 (T_1.1_: 45, T_1.2_: 50, T_1.3_: 50 C_1_:48; T_2.1_: 47, T_2.2_: 47, T_2.3_: 47, C_2_: 48)	1. Perimenopausal subgroup (irregular menstruation ≥ 3 months or amenorrhea < 12 months, FSH ≥ 10 IU/L) or early postmenopausal subgroup (amenorrhea between 1–5 years, FSH ≥ 20 IU/L) 2. Diagnostic criteria for menopausal symptom	≥ 14 times each week in the past 4 weeks	8 weeks	1) Dan Zhi Qing E formula granule (modified Qing E Tang, DZQE), 4.5 g 2) Er Zhi formula (EZ) granule, 2 g 3) Combined DZQE and EZ, 6.5 g, unspecified frequency	Placebo, NS	MENQOL (total, vasomotor, psychosocial, physical), hormone level (FSH, E_2_, testosterone), blood lipid profile, AE
**Grady et al. 2009 [29]**	University	217/217 (T_1_: 71, T_2_: 75, C: 71)	Postmenopausal	≥ 7/day moderate to severe hot flushes or 50/ week	12 weeks	MF 101 granule, 1) 5 g/day; 2) 10 g/day, one dose packed powder, twice/day	Placebo, one dose packed powder, twice/day	Frequency and severity of hot flushes (diary), MENQOL, Female Sexual Function Index, blood pressure, heart rate, breast and pelvic physical examination, endometrial double wall thickness, AE
**Haines et al. 2008 [30]**	Clinic of hospital	100 (T: 50, C: 50)/84 (T: 45, C: 39)	FSH > 18 IU/L, LH > 12.6 IU/L, E_2_ < 361 pmol/L; women with amenorrhea for ≥ 12 months.	Symptomatic	24 weeks	Dang Gui Bu Xue Tang capsule, 6 capsules (3g)/day	Placebo, 6 capsules/day	Frequency of hot flushes with different severity, night sweats (daily diary), MENQOL (vasomotor, psychosocial, physical, sexual), AE
**Nedeljkovic et al. 2014 [31]**	Hospital of university	20 (T: 10, C: 10)/19 (T: 10, C: 9)	Amenorrhea ≥ 12 months or had hysterectomy, initial score of ≥ 20 points on the MRS II, FSH > 30 IU/L	MHF ≥ 1 year, ≥ 20 MHF per week	12 weeks	Zhi Mu 14 capsule, 3 capsules, twice/day	Placebo, 3 capsules, twice/day	Severity and frequency of hot flushes, MRS II score (total, psychological, somatic, urogenital), AE
**Plotnikoff et al. 2011 [32]**	University	178/178 (T_1_: 57, T_2_: 62, C: 59)	Amenorrhea ≥ 12 months; previous hysterectomy without oophorectomy, FSH ≥ 40 mIU/mL, E_2_ ≤ 20 pg/mL; 2 months past surgery for hysterectomy with oophorectomy.	Mayo Hot Flash score ≥ 28/week	3 months	Gui Zhi Fu Ling Wan tablet, 1) low dose 7.5 g/day; 2) high dose 12.5 g/day, 5 tablets, twice/day	Placebo, 5 tablets, twice/day	Frequency and severity of hot flushes (daily diary), GCL, Sleep quality (PSQI), liver function tests, complete blood counts, hormone, AE
**van der Sluijs et al. 2009 [33]**	Clinical research center, clinics, university	92/92 (T: 46, C: 46)	Amenorrhea ≥ 12 months, FSH ≥ 40 mIU/mL, E_2_ ≤ 80 pg/mL	Average 5 vasomotor symptoms per day (~ 35/ week)	16 weeks	modified Er Xian Tang + Zhi Bai Di Huang Wan + black cohosh tablet, 2 tablets, twice/day	Placebo, 2 tablets, twice/day	Frequency, severity and score of hot flushes, GCL [total, psychological, anxiety, depression, somatic, vasomotor, sex], HFRDI total Scale, AE
**Xia et al. 2012 [34]**	Hospital of university	72 (T: 36, C: 36)/64 (T: 32, C: 32)	Perimenopausal: menstrual irregularity or amenorrhea for 3–11 months	≥ 14 hot flushes per week	8 weeks	modified Qing E Fang granule, 1 package (3.5g), once/day	Placebo, 1 package, once/day	MENQOL, hot flushes score (daily diary), frequency of night sweats, hormone (triglycerides (TG)), AE
**Zhong et al. 2013 [35]**	Hospital	108/108 (T: 54, C: 54)	Irregular menstrual cycles ≥ 3 months or amenorrhea ≥ 3 months within the previous 12 months	Experiencing hot flushes, MRS ≥ 28	12 weeks	Er Xian Tang granule, 15g a sachet, twice/day	Placebo, a sachet, twice/day	MENQOL, frequency and severity of hot flushes and hot flush scores (daily diary), hormone (FSH, LH, E_2_, progesterone), blood pressure, AE
**Zhou et al. 2007 [36]**	Hospital of university	69/69 (T: 36, C: 33)	Ovariectomy and met the diagnostic criteria for menopausal symptoms (clinical and laboratory examination)	Menopausal symptoms in the preceding 2 years	12 weeks	Geng Nian An granule, 1 sachet, twice/day	Placebo, 1 sachet, twice/day	Modified KI scale, hormone (FSH, LH, E_2_), MI, AE

Abbreviations: AE, adverse events; C, control group; E_2_, estradiol; EoT, end of treatment; FSH, follicle-stimulating hormone; GCL, Greene climacteric scale; HFRDI, hot flash related daily interference; HRT, hormone replacement therapy; KI, Kupperman index; LH, luteinizing hormone; MENQOL, menopause-specific quality of life; MI, maturation index of vaginal exfoliative cells; MRS, Menopause Rating Scale; NS: not specified; PSQI, Pittsburgh sleep quality index; T, treatment group.

### Methodological quality

**Fig 2:**
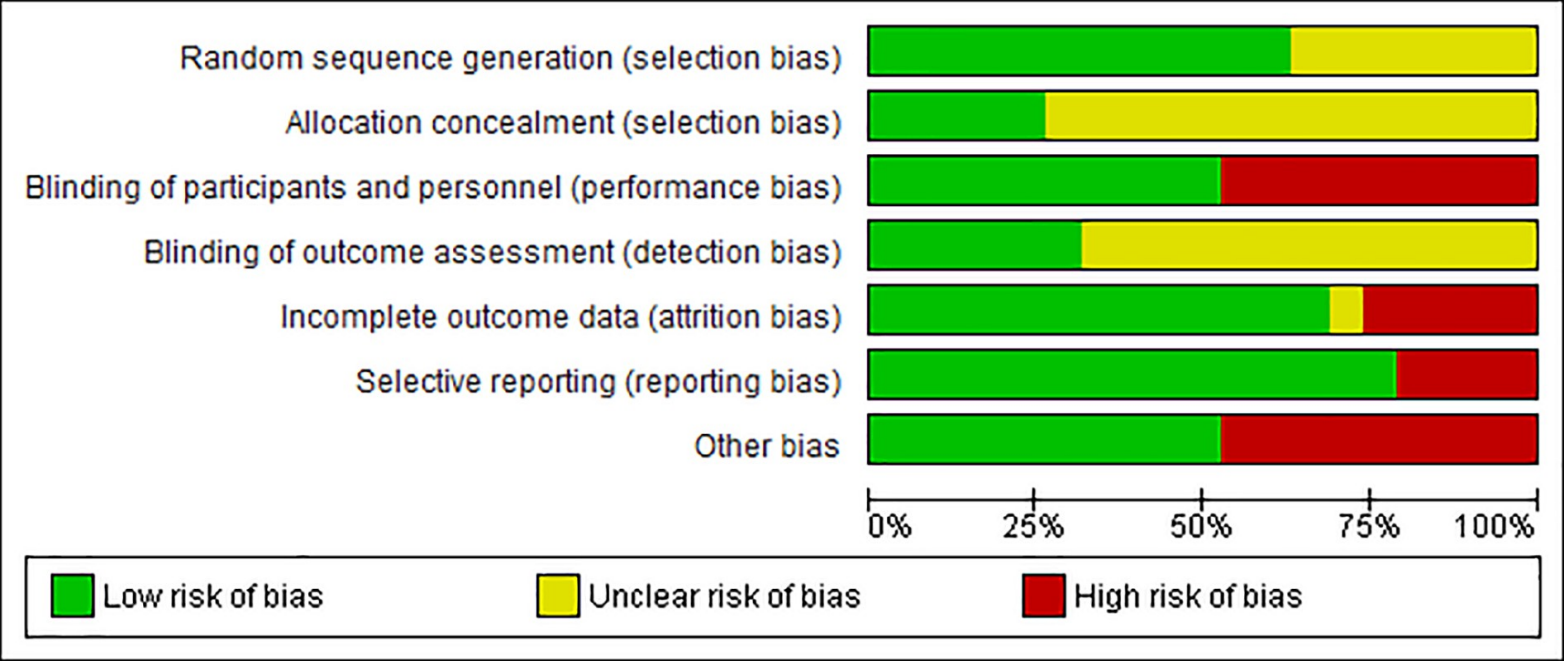
Risk of bias graph: Review authors' judgements about each risk of bias item presented as percentages across all included studies.

**Fig 3:**
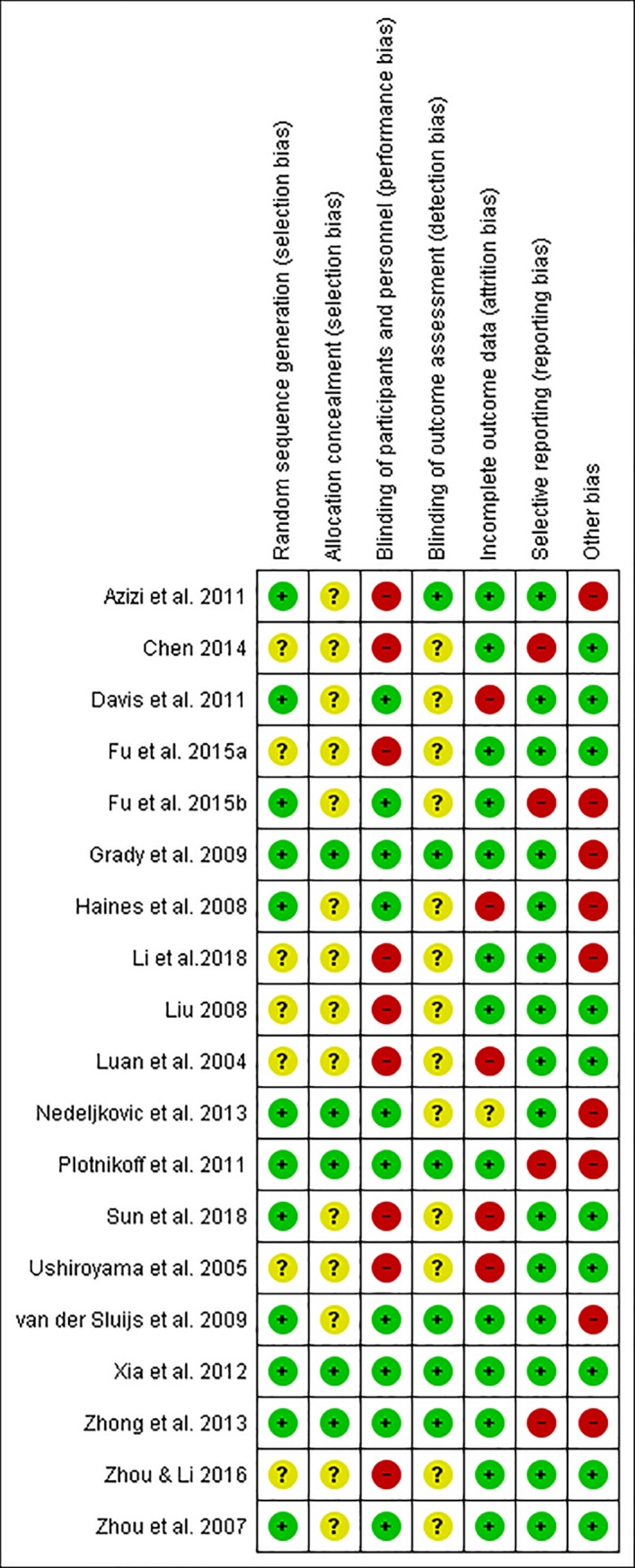
Risk of bias summary: Review authors' judgements about each risk of bias item for each included study.

More than 50% included studies had low risks of bias in the domains of selection (random sequence generation), performance, attrition, reporting bias and other bias (baseline and funding). Seven studies did not specify the randomization method [19–23, 25, 35], and the rest provided appropriate randomization methods (e.g. Random number table method). Five studies provided adequate information on how to allocate randomization numbers to participants and achieved a low risk of bias on the allocation concealment [29, 31, 32, 34, 35]. Ten studies did not achieve blinding due to different forms of interventions used in two groups (e.g. decoction vs tablet, capsule vs tablet) [18–26, 28]. Six studies blinded outcome assessors [18, 29, 32–35]; the rest either used self-reported questionnaires or provided insufficient information on the blinding of outcome assessors. Potential attrition bias was noticed in six studies [23–25, 27, 30, 31]. Three articles reported protocol but failed to report findings for all the outcome measures described in the protocol [28, 32, 35]. The rest 16 articles did not publish protocols. The outcomes described in the results section matched those provided in the methods section in all 15 studies except one [19]. Five studies had imbalanced baseline data but did not address in data analyses [18, 21, 28, 30, 35]. Four studies investigated the herbal formulae supplied by drug manufacturers [29, 31–33]. Thus, these nine studies may be associated with other biases. The assessment of methodological quality is summarized and presented in Figs 2 and 3.

#### Clinical effects

One study reported data from four arms: three treatment groups (Danzhiqing’e, Erzhi and combined formula) and one control group [26]. As a significantly imbalanced baseline of E_2_ between two treatment groups (Danzhiqing’e and Erzhi) and placebo was identified through baseline data analysis, data of these two comparisons were invalid and were excluded in the data analysis. Thus, only data from the treatment group of combined formula and the control group in this study were analyzed in this review.

#### CHM versus HRT

Nine of the included articles compared CHM with HRT [18–26]. Seven of them reported subjective measures [18–22, 24, 26] and five reported objective measures including relevant hormone level and peripheral blood flow [18, 19, 21, 23, 25].

CHM showed no significant differences in the effectiveness rate of MHF compared to HRT (OR 0.73, 95% CI 0.16 to 3.46) [19], but the effects on the MHF score favored HRT group (MD 1.00, 95% CI 0.45 to 1.55) [24].

Two studies measured vasomotor symptoms. One study indicated that CHM had a similar effectiveness rate of vasomotor symptoms to HRT (OR 1.25, 95% CI 0.34 to 4.59) [22]. However, the other study demonstrated that the vasomotor domain of KI scores favored CHM (MD -0.43, 95% CI -0.55 to -0.31) [20].

**Fig 4:**
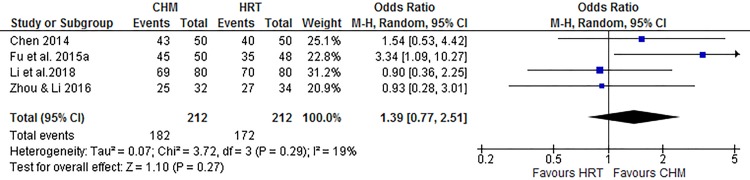
Meta-analysis of the total effectiveness rate of Chinese herbal medicine compared to menopausal hormone therapy for the treatment of menopausal hot flushes.

**Fig 5:**
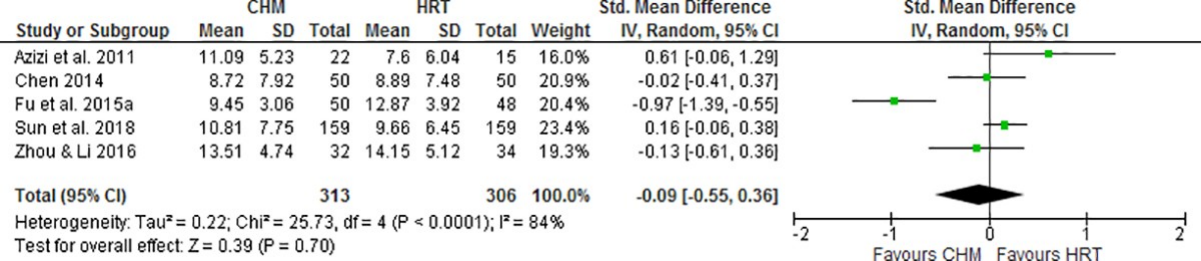
Meta-analysis of the total KI score of Chinese herbal medicine compared to menopausal hormone therapy for the treatment of menopausal hot flushes.

Six studies reported the menopausal symptoms in the aspect of the total effectiveness rate, KI score and QoL [18–21, 24, 26]. The pooled data indicated CHM has no significantly different total effectiveness rate when compared to HRT (OR 1.41, 95% CI 0.84 to 2.35, I^2^ = 19%, Fig 4) [19–21, 26], total KI score (SMD -0.09, 95% CI -0.55 to 0.36, I^2^ = 84%, Fig 5) [18–20, 24, 26] and QoL (MD -1.05, 95% CI -5.24 to 3.14) [24]. However, the results of the number of menopausal symptoms favored HRT group (MD 1.74, 95% CI 0.17 to 3.31) [18].

Data analyses demonstrated that two studies had a significant imbalance at baseline, one study for the FSH and E_2_ [18], and the other are for the E_2_ and LH [21]. Therefore, the results of these outcome measures were not included in meta-analysis. After the invalid data were removed from these two studies, results indicated that CHM had similar effects to HRT on the FSH level (MD -0.91, 95% CI -5.25 to 3.42, I^2^ = 0%) [21, 23], but the results of the E_2_ level were higher in HRT group (MD -23.28, 95% CI -33.51 to -13.04, I^2^ = 0%) [19, 23]. One study revealed a significant reduction of blood flow in the jaw and fingertips in CHM group compared to HRT (MD -3.56, 95% CI -5.17 to -1.95 under the jaw, MD -7.10, 95% CI -10.99 to -3.21 in the fingertip) which indicated CHM had a stronger inhibition of peripheral blood flow in the upper body [25].

#### CHM versus placebo

The other 10 RCTs compared CHM with placebo [27–36]. However, three of them were not included in the analysis due to lack of data [27, 29, 32]. All of the rest seven studies assessed subjective measures whereas only three of them evaluated objective measures including relevant hormone level [28, 35, 36].

Three different measures of MHF relief were used in the included studies, namely severity, frequency and score. Three studies reported MHF relief [30, 34, 35] and two reported changes of MHF relief [31, 33]. One study had significantly imbalanced MHF scores at the baseline so that it was not included in the analysis [35]. Data analysis results indicated a significant reduction of MHF severity in the CHM group compared to placebo group (MD -0.70, 95% CI-1.00 to -0.40) [35], but not significant in MHF frequency (SMD -0.06, 95% CI -0.26 to 0.15, I^2^ = 15%) [30, 35] or score (MD -0.64, 95% CI -2.17 to 0.89) [34]. Additionally, no significant differences of the changes in MHF relief were detected between CHM and placebo: frequency (SMD 0.02, 95% CI -0.35 to 0.40, I^2^ = 36%) [31, 33], severity (MD -0.49, 95% CI -1.41 to 0.43) [31], and score (MD 0.09, 95% CI -0.33 to 0.50) [33].

**Fig 6:**
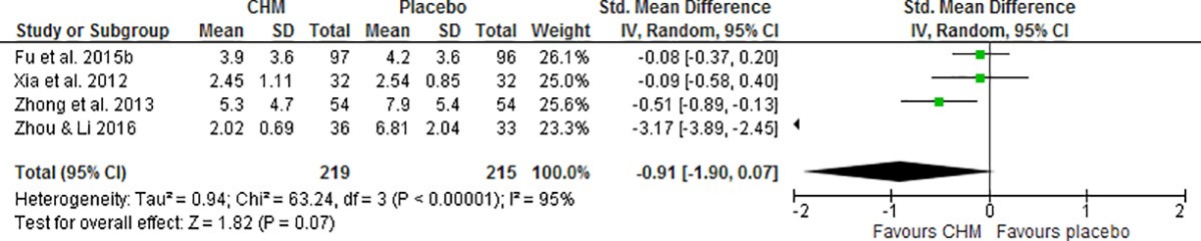
Meta-analysis of the vasomotor domain score of quality of life of Chinese herbal medicine compared to placebo for the treatment of menopausal hot flushes.

Six studies reported the score of vasomotor domains in the QoL questionnaire [28, 30, 33–36]. The synthesized data indicated CHM group showed no significant difference in the vasomotor score (SMD -0.91, 95% CI -1.90 to 0.07, I^2^ = 95%, Fig 6) [28, 34–36] or the changes of vasomotor scores (SMD 0.21, 95% CI -0.08 to 0.51) [30, 33] compared to that in placebo group at the end of treatment period.

Included studies also assessed QoL [28, 35], changes of QoL [31, 33] and total KI score [36]. The findings indicated that CHM significantly improved the total KI score (MD -12.61, 95% CI -15.21 to -10.01) [36], but not in QoL (SMD -0.33, 95% CI -0.83 to 0.17, I^2^ = 77%) [28, 35] or changes of QoL (SMD 0.08, 95% CI -0.20 to 0.46, I^2^ = 0%) [31, 33].

Three studies measured the E_2_ and FSH levels at the end of treatment [28, 35, 36] and two of them also measured the LH level [35, 36]. There were no statistically significant differences in hormone levels between CHM and placebo groups (Table 2).

**Table 2:** Data analyses of hormone levels between Chinese herbal medicine and placebo groups at baseline and at the end of treatment.

Hormone	Intervention	CHM vs placebo at baseline (MD [95% CI])	CHM vs placebo at the end of treatment (MD [95% CI])
Estradiol (E_2_)	Combined formula [28]	-15.88 [-41.69, 9.93]	73.32 [44.30, 102.34] *
	Er Xian decoction [35]	-76.10 [-177.12, 24.92]	16.30 [-133.14, 165.74]
	Geng Nian An [36]	0.30 [-2.59, 3.19]	11.40 [7.82, 14.98]*
	Total		37.92 [-16.75, 92.59], I^2^ = 88%
Follicle-stimulating hormone (FSH)	Dan Zhi Qing E formula [28]	-2.06 [-9.91, 5.79]	1.16 [-6.03, 8.35]
	Er Zhi formula [28]	-1.58 [-8.73, 5.57]	-1.06 [-9.45, 7.33]
	Combined formula [28]	0.08 [-7.62, 7.78]	1.60 [-5.46, 8.66]
	Er Xian decoction [35]	13.80 [-0.49, 28.09]	16.20 [1.67, 30.73]
	Geng Nian An [36]	0.60 [-4.95, 6.15]	-12.30 [-17.46, -7.14]*
	Total		0.32 [-13.79, 14.43], I^2^ = 89%
Luteinizing hormone (LH)	Er Xian decoction [35]	2.90 [-3.06, 8.86]	4.90 [-0.67, 10.47]
	Geng Nian An [36]	0.50 [-2.81, 3.81]	-9.80 [-13.16, -6.44]*
	Total		-2.62 [-17.03, 11.78], I^2^ = 95%

Notes

*, significant difference favored CHM group. Abbreviations: CHM, Chinese herbal medicine; CI, confidence intervals; MD, mean difference.

#### Safety

Two out of the 19 included studies did not provide the safety data of interventions [19, 25]. Five studies indicated that no AEs were reported in the CHM group [18, 20, 21, 26, 36]. One study reported CHM had much fewer AEs than HRT [22]. Five trials stated that no statistically significant differences in AEs were observed between CHM and control groups [27, 30, 31, 33, 35]. The rest studies reported AEs from each arm without a statement of comparison between both arms. The most commonly seen AEs were gastrointestinal symptoms (such as loose stools, stomach discomfort/ache, nausea) (93/1010) and breast tenderness (31/229) (S3 Table).

#### Sensitivity analyses and publication bias

Due to the limited number of included studies, sensitivity analysis could not be performed in this review.

Four RCTs compared CHM and HRT as well as nine studies compared CHM with placebo involved more than one study, thus their Rosenthal Failsafe-Ns were assessed for publication bias. Most outcomes presented a Failsafe-N at 0 including total effectiveness rate, total KI score, FSH level when comparing CHM with HRT; MHF frequency, changes in MHF frequency, changes of vasomotor scores, changes of QoL and FSH when comparing CHM with placebo. Other Failsafe-Ns are summarized in Table 3.

**Table 3:** Other Rosenthal Failsafe-N for publication bias in outcome measures included more than one studies.

Outcomes	Comparisons	Studies	Rosenthal Failsafe-N
**Estradiol**	CHM versus HRT	[19, 23]	7
**Vasomotor score**	CHM versus placebo	[28, 34–36]	53
**Quality of life**	CHM versus placebo	[28, 35]	4
**Estradiol**	CHM versus placebo	[35, 36]	38
**Luteinizing hormone**	CHM versus placebo	[35, 36]	3

Abbreviation: CHM, Chinese herbal medicine.

#### Frequency of CHM formulae and herbs

**Fig 7:**
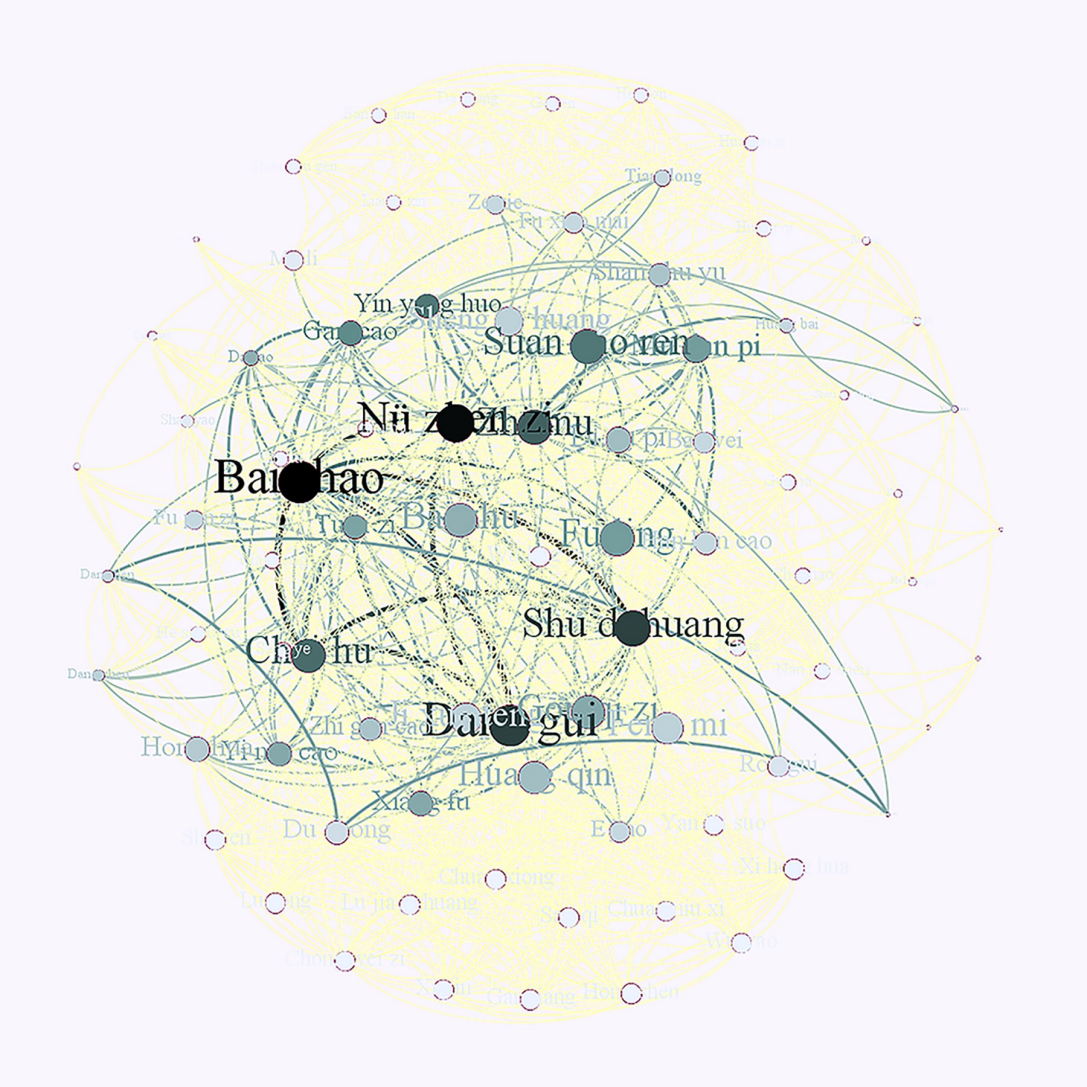
The network of herb pairs in the prescribed CHM formulae from included studies.

Among the included studies, 18 formulae were prescribed to the participants (S2 Table). Two formulae were investigated in more than one study, namely Kun Tai capsule [19, 23, 24] and Gui Zhi Fu Ling Wan [25, 32]. Other formulae were six classic CHM formulae [18, 21, 28, 30, 35], five newly developed formulae [20, 26, 29, 31, 36], four modified formulae [22, 27, 33, 34], and one combined formula [28]. The top 10 frequently ranked individual herbs in the included studies were Bai shao (Paeoniae Radix Alba, *Paeonia lactiflora* Pall.) (n = 12), Shu di huang (Rehmanniae Radix Praeparata, *Rehmannia glutinosa* Libosch.) (n = 9), Nü zhen zi (Ligustri Lucidi Fructus, *Ligustrum lucidum* Ait.) (n = 9), Fu ling (Poria, *Poria cocos* (Schw.) Wolf) (n = 8), Dang gui (Angelicae Sinensis Radix, *Angelica sinensis* (Oliv.) Diels) (n = 8), Zhi mu (Anemarrhenae Rhizoma, *Anemarrhena asphodeloides* Bge.) (n = 7), Chai hu (Bupleuri Radix, *Bupleurum chinense* DC. or *Bupleurum scorzonerifolium* Willd.) (n = 6), Mu dan pi (Moutan Cortex, *Paeonia suffruticosa* Andr.) (n = 6), Huang qin (Scutellariae Radix, *Scutellaria baicalensis* Georgi) (n = 5), Suan zao ren (Ziziphi Spinosae Semen, *Ziziphus jujuba* Mill. var. spinosa (Bunge) Hu ex H. F. Chou) (n = 5), and Yin yang huo (Epimedii Folium, *Epimedium brevicornum* Maxim., *Epimedium sagittatum* (Sieb. et Zucc.) Maxim., *Epimedium pubescens* Maxim. or *Epimedium koreanum* Nakai) (n = 5). The network of herb pairs in the prescribed CHM formulae from included studies is presented in Fig 7. The top three frequently used herb pairs in the prescribed formulae were Chai hu plus Bai shao, Bai shao plus Shu di huang, and Bai shao combined with Dang gui (n = 6). The following pairs were Chai hu plus Shu di huang, Chai hu plus Dang gui, Bai shao plus Nü zhen zi, and Dang gui plus Nü zhen zi (n = 5).

## Discussion

MHF is one of the menopausal symptoms which occur before, during and after menopause transition [2]. CHM offers more options to MHF management in patients with concerns about HRT, but more evidence was warranted [8, 9]. This study aimed to evaluate the therapeutic effects and safety of CHM formulae.

More than half of included studies had low risks of bias in random sequence generation, blinding of participants and personnel, attrition, reporting and baseline comparison. After removing the studies with imbalanced baseline, the findings of this review suggested that CHM had similar effects to HRT in the effectiveness rate (MHF, vasomotor symptoms and menopausal symptoms), total KI score, QoL and FSH level. CHM could significantly improve the score of vasomotor domains of QoL and upper-body peripheral blood flow (jaw and fingertips) compared to HRT. CHM also significantly reduced the severity of MHF and total KI score compared to the placebo group, but with no significant difference in hormone levels. Although the majority of the studies measured MHF relief and QoL as the primary outcomes, different measures were used across studies, which led to difficulty in data synthesis. Most of the studies reported findings at the end of treatment, whilst a few reported the changes from baseline [30, 31, 33] which could not be synthesized with other results. Additionally, several different scales were utilized to measure QoL in the identified studies including Greene Climacteric Scale, Green Scale score, Hot Flash Related Daily Interference, Menopause-Specific Quality of Life and Menopause Rating Scale. Application of a standardized QoL questionnaire may make the data synthesis and comparison across studies more feasible.

Positive clinical effects of CHM may be related to potential estrogen-like actions of the prescribed herbs. In the identified popularly prescribed herbs, Bai shao, Dang gui, Zhi mu, Chai hu, Huang qin and Yin yang huo have been found to contain phytoestrogens by gas chromatography-mass spectrometry [37]. Shu di huang, Bai shao and Dang gui were examined to have phytoestrogenic effects on Sprague Dawley rats and cultural MCF-7 cells [38]. Yin yang huo showed bidirectional adjustment of estrogenic effects (when inverted independently or together with diethylstilbestrol) in female Kunming mice. The direction of phytoestrogenic effects depended on the internal estrogen level [39]. The combination of Nü zhen zi and Han lian cao (Ecliptae Herba, *Eclipta prostrata* L.) demonstrated an effect in activating the estrogenic activity in an *in vitro* study using cultural MCF-7 cells line [40].

Another hypothesized specific vasodilator of MHF in humans, named CGRP, is responsible for peripheral circulation by affecting the signal transition of the perivascular nerves [41]. It significantly rises during MHF episodes [6]. Three of the top ten popular prescribed herbs, namely Bai shao, Mu dan pi and Fu ling, also ingredients of Gui Zhi Fu Ling Wan, were found to significantly reduce the plasma concentration of CGRP [42].

To the best of our knowledge, there is no published study which has reviewed CHM for MHF. Three studies reviewed CHM on menopausal symptoms in 2008, 2010 and 2012 [43–45] and one study reviewed CHM on drug-induced hot flushes in breast cancer patients [46]. The current review differs from them as it focused on patients with MHF and compared CHM with HRT or placebo only. Thus, a different set of RCTs from other reviews was included in our review. However, this review did not search databases in other languages, such as Japanese or Korean. Thus, there is a possibility that some studies related to CHM for MHF have not been identified.

CHM appears to be safe for females with MHF as AEs were few and mild. Overall, there is no noteworthy risk of intakes of CHM. As some herbs may have phytoestrogenic effects or estrogen-like effects, they should be used with caution when prescribed to patients with hormone-dependent conditions, such as breast cancer [46]. Further investigations of contraindications and possible herb-drug interactions are needed [28, 46].

## Conclusions

Noting the limitation of quality and quantity of the results, it is worth further exploring CHM as an alternative therapy to improve the MHF sufferers’ subjective feeling as well as the objective changes in peripheral blood flow. Due to the high variations of investigated CHM formulae in the included studies, future rigorously conducted studies investigating on a specific non-modified CHM formula for the management of MHF are recommended.

## Supporting information

S1 PRISMA checklist (DOCX)

S1 Table Search strategies used for electronic database search in PubMed. (DOCX)

S2 Table Ingredients of Chinese herbal formulae in the included studies. (DOCX)

S3 Table Details of adverse events reported in the included studies. (DOCX)
