# Supplementary material for: Chinese herbal formulae for the treatment of menopausal hot flushes: A systematic review and meta-analysis
Source: PLoS One. 2019 Sep 19;14(9):e0222383. doi: 10.1371/journal.pone.0222383 (PMC6752783; doi:10.1371/journal.pone.0222383)
Supplement: S1 Table — (DOCX) [file pone.0222383.s002.docx]

Supplementary material

## S1 Table. Search strategies used for electronic database search in PubMed

|  | Search | Query |
| --- | --- | --- |
| Intervention | #1 | Search herb |
|  | #2 | Search herbal medicine |
|  | #3 | Search Chinese medicine |
|  | #4 | Search traditional Chinese medicine |
|  | #5 | Search formula* |
|  | #6 | #1 OR #2 OR #3 OR #4 OR #5 |
| Condition | #7 | Search hot flash* |
|  | #8 | Search hot flush* |
|  | #9 | Search menopausal hot flush* |
|  | #10 | Search menopausal hot flash* |
|  | #11 | #7 OR #8 OR #9 OR #10 |
|  | #12 | #6 AND #11 |
