# Supplementary material for: Chinese herbal formulae for the treatment of menopausal hot flushes: A systematic review and meta-analysis
Source: PLoS One. 2019 Sep 19;14(9):e0222383. doi: 10.1371/journal.pone.0222383 (PMC6752783; doi:10.1371/journal.pone.0222383)
Supplement: S2 Table — (DOCX) [file pone.0222383.s003.docx]

Supplementary material

## S2 Table. Ingredients of Chinese herbal formulae in the included studies

| Study ID | Preparation | Formula name | Ingredients (pin yin name, pharmaceutical name, scientific name) |
| --- | --- | --- | --- |
| Azizi et al. 2011 [1] | Honey pill | Kun Bao Wan | Nü zhen zi (Ligustri Lucidi Fructus, *Ligustrum lucidum* Ait.), Chi shao (Paeoniae Radix Rubra, *Paeonia lactiflora* Pall. or *Paeonia veitchii* Lynch), Bai shao (Paeoniae Radix Alba, *Paeonia lactiflora* Pall.), Sheng di huang (Rehmanniae Radix, *Rehmannia glutinosa* Libosch.), Fu pen zi (Rubi Fructus, *Rubus chingii* Hu), Dang gui (Angelicae Sinensis Radix, *Angelica sinensis* (Oliv.) Diels), Tu si zi (Cuscutae Semen, *Cuscuta australis* R. Br. or *Cuscuta chinensis* Lam.), Ji xue teng (Kadsurae Caulis, *Kadsura interior* A. C. Smith), Gou qi zi (Lycii Fructus, *Lycium barbarum* L.), Suan zao ren (Ziziphi Spinosae Semen, *Ziziphus jujuba* Mill. var. spinosa (Bunge) Hu ex H. F. Chou), Huang qin (Scutellariae Radix, *Scutellaria baicalensis* Georgi), Ju hua (Chrysanthemi Flos, *Chrysanthemum morifolium* Ramat.), Han lian cao (Ecliptae Herba, *Eclipta prostrata* L.), Nan sha shen (Adenophorae Radix, *Adenophora tetraphylla* (Thunb.) Fisch. or *Adenophora stricta* Miq.), Sang ye (Mori Folium, *Morus alba* L.), Bai wei (Cynanchi Atrati Radix et Rhizoma, *Cynanchum atratum* Bge. or *Cynanchum versicolour* Bge.), Zhi mu (Anemarrhenae Rhizoma, *Anemarrhena asphodeloides* Bge.), He shou wu (Polygoni Multiflori Radix, *Polygonum multiflorum* Thunb.), Shi hu (Dendrobii Caulis, *Dendrobium nobile* Lindl., *Dendrobium chysotoxum* Lindl. or *Dendrobium fimbriatum* Hook.), Di gu pi (Lycii Cortex, *Lycium chinense* Mill.or *Lycium barbarum* L.), Gui jia (Testudinis Carapax et Plastrum, *Chinemys reevesii* (Gray)), Mai dong (Ophiopogonis Radix, *Ophiopogon japonicus* (L.f) Ker-Gawl.), Zhen zhu mu (Margaritifera Concha, *Hyriopsis cumingii* (Lea), *Cristaria plicata* (Leach) or *Pteria martensii* (Dunker)), Feng mi (honey, *Apis cerana* Fabricius or *Apis mellifera* Linnaeus.) |
| Chen 2014 [2] | Capsule | He Yan Kun Tai Capsule (modified Huang Lian E Jiao Tang) | Shu di huang (Rehmanniae Radix Praeparata, *Rehmannia glutinosa* Libosch.), Bai shao (Paeoniae Radix Alba, *Paeonia lactiflora* Pall.), E jiao (Asini Corii Colla, *Equus asinus* L.), Huang lian (Coptidis Rhizoma, *Coptis chinensis* Franch., Coptis deltoidea C. Y. Cheng et Hsiao or *Coptis teeta* Wall.), Huang qin (Scutellariae Radix, *Scutellaria baicalensis* Georgi), Fu ling (Poria, *Poria cocos* (Schw.) Wolf) |
| Davis et al. 2001 [3] | Granule | Modified Liu Wei Di Huang Wan | Shu di huang (Rehmanniae Radix Praeparata, *Rehmannia glutinosa* Libosch.), Shan zhu yu (Corni Fructus, *Cornus officinalis* Sieb. et Zucc.), Shan yao (Dioscoreae Rhizoma, *Dioscorea opposita* Thunb.), Ze xie (Alismatis Rhizoma, *Alisma orientalis* (Sam.) Juzep.), Mu dan pi (Moutan Cortex, *Paeonia suffruticosa* Andr.), Fu shen (Poriae Sclerotium Pararadicis, *Poria cocos* (Schw.) Wolf), Chen Pi (Citri Reticulatae Pericarpium, *Citrus reticulata* Blanco), Di gu pi (Lycii Cortex, *Lycium chinense* Mill.or *Lycium barbarum* L.), He huan pi (Albiziae Cortex, *Albizia julibrissin* Durazz.), Suan zao ren (Ziziphi Spinosae Semen, *Ziziphus jujuba* Mill. var. spinosa (Bunge) Hu ex H. F. Chou), Han lian cao (Ecliptae Herba, *Eclipta prostrata* L.), Nü zhen zi (Ligustri Lucidi Fructus, *Ligustrum lucidum* Ait.), |
| Fu et al. 2015a [4] | Decoction | Chinese herbal formula | Dang gui (Angelicae Sinensis Radix, *Angelica sinensis* (Oliv.) Diels), Chai hu (Bupleuri Radix, *Bupleurum chinense* DC. or *Bupleurum scorzonerifolium* Willd.), Gan cao (Glycyrrhizae Radix et Rhizoma, *Glycyrrhiza uralensis* Fisch., *Glycyrrhiza inflata* Bat., *Glycyrrhiza glabra* L.), Bai shao (Paeoniae Radix Alba, *Paeonia lactiflora* Pall.), Shu di huang (Rehmanniae Radix Praeparata, *Rehmannia glutinosa* Libosch.), Tu si zi (Cuscutae Semen, *Cuscuta australis* R. Br. or *Cuscuta chinensis* Lam.), Yin yang huo (Epimedii Folium, *Epimedium brevicornum* Maxim., *Epimedium sagittatum* (Sieb. et Zucc.) Maxim., *Epimedium pubescens* Maxim. or *Epimedium koreanum* Nakai), Nü zhen zi (Ligustri Lucidi Fructus, *Ligustrum lucidum* Ait.), Yi mu cao (Leonuri herba, *Leonurus japonicus* Houtt.), Xiang fu (Cyperi Rhizoma, *Cyperus rotundus* L.), Da zao (Jujubae Fructus, *Ziziphus jujuba* Mill.) |
| Fu et al. 2015b [5] | Granule | 1) Dan Zhi Qing E formula (DZQE, modified Qing E Wan) 2) Er Zhi formula (EZ) 3) DZQE+EZ | 1) Dan shen (Salviae Miltiorrhizae Radix et Rhizoma, *Salvia miltiorrhiza* Bge.), Zhi mu (Anemarrhenae Rhizoma, *Anemarrhena asphodeloides* Bge.), Du zhong (Eucommiae Cortex, *Eucommia ulmoides* Oliv.), Bu gu zhi (Psoraleae Fructus, *Psoralea corylifolia* L.) 2) Nü zhen zi (Ligustri Lucidi Fructus, *Ligustrum lucidum* Ait.), Han lian cao (Ecliptae Herba, *Eclipta prostrata* L.) 3) Dan shen (Salviae Miltiorrhizae Radix et Rhizoma, *Salvia miltiorrhiza* Bge.), Zhi mu (Anemarrhenae Rhizoma, *Anemarrhena asphodeloides* Bge.), Du zhong (Eucommiae Cortex, *Eucommia ulmoides* Oliv.), Bu gu zhi (Psoraleae Fructus, *Psoralea corylifolia* L.), Nü zhen zi (Ligustri Lucidi Fructus, *Ligustrum lucidum* Ait.), Han lian cao (Ecliptae Herba, *Eclipta prostrata* L.) |
| Grady et al. 2009 [6] | Granule | MF 101 | Ban zhi lian (Scutellariae Barbatae Herba, *Scutellaria barbata* D. Don), Shan dou gen (Sophorae Tonkinensis Radix et Rhizoma*, Sophora tonkinensis* Gagnep.), Zhi mu (Anemarrhenae Rhizoma, *Anemarrhena asphodeloides* Bge.), Hei dou (Sojae Semen Nigrum, *Glycine max* (L.) Merr.), Gan cao (Glycyrrhizae Radix et Rhizoma, *Glycyrrhiza uralensis* Fisch., *Glycyrrhiza inflata* Bat. or *Glycyrrhiza glabra* L.), Da huang (Rhei Radix et Rhizoma, *Rheum palmatum* L., *Rheum tanguticum* Maxim. ex Balf., or *Rheum officinale* Baill.), Fu xiao mai (Tritici Fructus Levis, *Triticum aestivum* L.), Huang qi (Astragali Radix, *Astragalus membranaceus* (Fisch.) Bge. var. mongholicus (Bge.) Hsiao or *Astragalus membranaceus* (Fisch.) Bge.), Sheng di huang (Rehmanniae Radix, *Rehmannia glutinosa* Libosch.), Nü zhen zi (Ligustri Lucidi Fructus, *Ligustrum lucidum* Ait.), Suan zao ren (Ziziphi Spinosae Semen, *Ziziphus jujuba* Mill. var. spinosa (Bunge) Hu ex H. F. Chou), Lian zi xin (Nelumbinis Plumula, *Nelumbo nucifera* Gaertn.), Fu ling (Poria, *Poria cocos* (Schw.) Wolf), Ze xie (Alismatis Rhizoma, *Alisma orientalis* (Sam.) Juzep.), Mu dan pi (Moutan Cortex, *Paeonia suffruticosa* Andr.), Shan zhu yu (Corni Fructus, *Cornus officinalis* Sieb. et Zucc.), Niu xi (Achyranthis Bidentatae Radix, *Achyranthes bidentata* Bl.), Mu li (Ostreae Concha, *Ostrea gigas* Thunberg, *Ostrea talienwhanensis* Crosse or *Ostrea rivularis* Gould), Tian dong (Asparagi Radix, *Asparagus cochinchinensis* (Lour.) Merr.), Ge gen (Puerariae Lobatae Radix, *Pueraria lobata* (Willd.) Ohwi), Bai zhu (Atractylodis Macrocephalae Rhizoma, *Atractylodes macrocephala* Koidz.), Yin yang huo (Epimedii Folium, *Epimedium brevicornum* Maxim., *Epimedium sagittatum* (Sieb. et Zucc.) Maxim., *Epimedium pubescens* Maxim. or *Epimedium koreanum* Nakai) |
| Haines et al. 2008 [7] | Capsule | Dang Gui Bu Xue Tang | Dang gui (Angelicae Sinensis Radix, *Angelica sinensis* (Oliv.) Diels), Huang qi (Astragali Radix, *Astragalus membranaceus* (Fisch.) Bge. var. mongholicus (Bge.) Hsiao or *Astragalus membranaceus* (Fisch.) Bge.) |
| Li et al.  2018 [8] | Honey pill | Ding Kun Dan | Hong shen (Ginseng Radix et Rhizoma Rubra, *Panax ginseng* C. A. Mey.), Lu rong (Cervi cornus pantotrichum, *Cervus nippon* Temminck or Cervus elaphus Linnaeus), Xi hong hua (Croci Stigma, *Crocus sativus* L.), Ji xue teng (Kadsurae Caulis, *Kadsura interior* A. C. Smith), San qi (Notoginseng Radix et Rhizoma, *Panax notoginseng* (Burk.) F.H.Chen), Bai shao (Paeoniae Radix Alba, *Paeonia lactiflora* Pall.), Shu di huang (Rehmanniae Radix Praeparata, *Rehmannia glutinosa* Libosch.), Dang gui (Angelicae Sinensis Radix, *Angelica sinensis* (Oliv.) Diels), Bai zhu (Atractylodis Macrocephalae Rhizoma, *Atractylodes macrocephala* Koidz.), Gou qi zi (Lycii Fructus, *Lycium barbarum* L.), Huang qin (Scutellariae Radix, *Scutellaria baicalensis* Georgi), Xiang fu (Cyperi Rhizoma, *Cyperus rotundus* L.), Chong wei zi (Leonuri Fructus, *Leonurus japonicus* Houtt.), Chuan xiong (Chuanxiong Rhizoma, *Ligusticum chuanxiong* Hort.), Lu jiao shuang (Cervi Cornu Degelatinatum, *Cervus elaphus* Linnaeus or *Cervus nippon* Temminck), E jiao (Asini Corii Colla, *Equus asinus* L.), Yan hu suo (Corydalis Rhizoma, *Corydalis yanhusuo* W.T. Wang), Hong hua (Carthami Flos, *Carthamus tinctorius* L.), Yi mu cao (Leonuri herba, *Leonurus japonicus* Houtt.), Wu ling zhi (Faeces Trogopterori), Fu ling (Poria, *Poria cocos* (Schw.) Wolf), Chai hu (Bupleuri Radix, *Bupleurum chinense* DC. or *Bupleurum scorzonerifolium* Willd.), Wu yao (Linderae Radix, *Lindera aggregata* (Sims) Kosterm.), Sha ren (Amomi Fructus, *Amomum villosum* Lour., *Amomum villosum* Lour. var. xanthioides T. L. Wu et Senjen or *Amomum longiligulare* T. L. Wu), Du zhong (Eucommiae Cortex, *Eucommia ulmoides* Oliv.), Gan jiang (Zingiberis Rhizoma, *Zingiber officinale* Rosc.), Xi xin (Asari Radix et Rhizoma, *Asarum heterotropoides* Fr. Schmidt var. mandshuricum (Maxim) Kitag., *Asarum sieboldii* Miq. var. seoulense Nakai or *Asarum sieboldii* Miq.), Chuan niu xi (Cyathulae Radix, *Cyathula officinalis* Kuan), Rou gui (Cinnamomi Cortex, *Cinnamomum cassia* Presl), Zhi gan cao (Glycyrrhizae Radix et Rhizoma Praeparata cum Melle, *Glycyrrhiza uralensis* Fish., *Glycyrrhiza inflata* Bat. or *Glycyrrhiza glabra* L.), Feng mi (honey, *Apis cerana* Fabricius or *Apis mellifera* Linnaeus.) |
| Liu 2008 [9] | Decoction | Modified Dan Zhi Xiao Yao San | Mu dan pi (Moutan Cortex, *Paeonia suffruticosa* Andr.), Zhi zi (Gardeniae Fructus, *Gardenia jasminoides* Ellis), Chai hu (Bupleuri Radix, *Bupleurum chinense* DC. or *Bupleurum scorzonerifolium* Willd.), Dang gui (Angelicae Sinensis Radix, *Angelica sinensis* (Oliv.) Diels), Bai shao (Paeoniae Radix Alba, *Paeonia lactiflora* Pall.), Fu ling (Poria, *Poria cocos* (Schw.) Wolf), Bai zhu (Atractylodis Macrocephalae Rhizoma, *Atractylodes macrocephala* Koidz.), Zhi gan cao (Glycyrrhizae Radix et Rhizoma Praeparata cum Melle, *Glycyrrhiza uralensis* Fish., *Glycyrrhiza inflata* Bat. or *Glycyrrhiza glabra* L.), Shan zhu yu (Corni Fructus, *Cornus officinalis* Sieb. et Zucc.), Gou qi zi (Lycii Fructus, *Lycium barbarum* L.), Nü zhen zi (Ligustri Lucidi Fructus, *Ligustrum lucidum* Ait.), Shu di huang (Rehmanniae Radix Praeparata, *Rehmannia glutinosa* Libosch.) |
| Luan et al. 2004 [10] | Capsule | He Yan Kun Tai Capsule (modified Huang Lian E Jiao Tang) | Shu di huang (Rehmanniae Radix Praeparata, *Rehmannia glutinosa* Libosch.), Bai shao (Paeoniae Radix Alba, *Paeonia lactiflora* Pall.), E jiao (Asini Corii Colla, *Equus asinus* L.), Huang lian (Coptidis Rhizoma, *Coptis chinensis* Franch., Coptis deltoidea C. Y. Cheng et Hsiao or *Coptis teeta* Wall.), Huang qin (Scutellariae Radix, *Scutellaria baicalensis* Georgi), Fu ling (Poria, *Poria cocos* (Schw.) Wolf) |
| Nedeljkovic et al. 2014 [11] | Capsule | Zhi Mu 14 (modified Gan Mai Da Zao Tang and Qing Hao Bie Jia Tang) | Shu di huang (Rehmanniae Radix Praeparata, *Rehmannia glutinosa* Libosch.), Zhi mu (Anemarrhenae Rhizoma, *Anemarrhena asphodeloides* Bge.), Suan zao ren (Ziziphi Spinosae Semen, *Ziziphus jujuba* Mill. var. spinosa (Bunge) Hu ex H. F. Chou), Bai wei (Cynanchi Atrati Radix et Rhizoma, *Cynanchum atratum* Bge. or *Cynanchum versicolour* Bge.), Gan cao (Glycyrrhizae Radix et Rhizoma, *Glycyrrhiza uralensis* Fisch., *Glycyrrhiza inflata* Bat. or *Glycyrrhiza glabra* L.), Shou wu teng (Polygoni Multiflori Caulis, *Polygonum multiflorum* Thunb.), Bai shao (Paeoniae Radix Alba, *Paeonia lactiflora* Pall.), Di gu pi (Lycii Cortex, *Lycium chinense* Mill.or *Lycium barbarum* L.), Huang bai (Phellodendri Chinensis Cortex, *Phellodendron chinense* Schneid.), Fu xiao mai (Tritici Fructus Levis, *Triticum aestivum* L.), Mu dan pi (Moutan Cortex, *Paeonia suffruticosa* Andr.), Qing hao (Artemisiae Annuae Herba, *Artemisia annua* L.), Chai hu (Bupleuri Radix, *Bupleurum chinense* DC. or *Bupleurum scorzonerifolium* Willd.), Da zao (Jujubae Fructus, *Ziziphus jujuba* Mill.) |
| Plotnikoff et al. 2011 [12] | Tablet | Gui Zhi Fu Ling Wan (TU-025) | Rou gui (Cinnamomi Cortex, *Cinnamomum cassia* Presl), Shao yao (Paeoniae Radix Alba, *Paeonia lactiflora* Pall.), Tao ren (Persicae Semen, *Prunus persica* (L.) Batsch or *Prunus davidiana* (Carr.) Franch.), Fu ling (Poria, *Poria cocos* (Schw.) Wolf), Mu dan pi (Moutan Cortex, *Paeonia suffruticosa* Andr.) |
| Sun et al. 2018 [13] | Capsule | He Yan Kun Tai Capsule (modified Huang Lian E Jiao Tang) | Shu di huang (Rehmanniae Radix Praeparata, *Rehmannia glutinosa* Libosch.), Bai shao (Paeoniae Radix Alba, *Paeonia lactiflora* Pall.), E jiao (Asini Corii Colla, *Equus asinus* L.), Huang lian (Coptidis Rhizoma, *Coptis chinensis* Franch., Coptis deltoidea C. Y. Cheng et Hsiao or *Coptis teeta* Wall.), Huang qin (Scutellariae Radix, *Scutellaria baicalensis* Georgi), Fu ling (Poria, *Poria cocos* (Schw.) Wolf) |
| Ushiroyama et al. 2005 [14] | Powder | Gui Zhi Fu Ling Wan | Rou gui (Cinnamomi Cortex, *Cinnamomum cassia* Presl), Shao yao (Paeoniae Radix Alba, *Paeonia lactiflora* Pall.), Tao ren (Persicae Semen, *Prunus persica* (L.) Batsch or *Prunus davidiana* (Carr.) Franch.), Fu ling (Poria, *Poria cocos* (Schw.) Wolf), Mu dan pi (Moutan Cortex, *Paeonia suffruticosa* Andr.) |
| van der Sluijs et al. 2009 [15] | Tablet | modified Er Xian Tang and Zhi Bai Di Huang Wan | Suan zao ren (Ziziphi Spinosae Semen, *Ziziphus jujuba* Mill. var. spinosa (Bunge) Hu ex H. F. Chou), Sheng di huang (Rehmanniae Radix, *Rehmannia glutinosa* Libosch.), Zhi mu (Anemarrhenae Rhizoma, *Anemarrhena asphodeloides* Bge.), Tian dong (Asparagi Radix, *Asparagus cochinchinensis* (Lour.) Merr.), Yin yang huo (Epimedii Folium, *Epimedium brevicornum* Maxim., *Epimedium sagittatum* (Sieb. et Zucc.) Maxim., *Epimedium pubescens* Maxim. or *Epimedium koreanum* Nakai), Xian mao (Curculiginis Rhizoma, *Curculigo orchioides* Gaertn.), Huang bai (Phellodendri Chinensis Cortex, *Phellodendron chinense* Schneid.), Hei sheng ma (Black cohosh, *Cimicifuga racemosa*) |
| Xia et al. 2012 [16] | Granule | modified Qing E Fang | Du zhong (Eucommiae Cortex, *Eucommia ulmoides* Oliv.), Bu gu zhi (Psoraleae Fructus, *Psoralea corylifolia* L.), Dan shen (Salviae Miltiorrhizae Radix et Rhizoma, *Salvia miltiorrhiza* Bge.) |
| Zhong et al. 2013 [17] | Granule | Er Xian Tang | Xian mao (Curculiginis Rhizoma, *Curculigo orchioides* Gaertn.), Yin yang huo (Epimedii Folium, *Epimedium brevicornum* Maxim., *Epimedium sagittatum* (Sieb. et Zucc.) Maxim., *Epimedium pubescens* Maxim. or *Epimedium koreanum* Nakai), Ba ji tian (Morindae Officinalis Radix, *Morinda officinalis* How), Dang gui (Angelicae Sinensis Radix, *Angelica sinensis* (Oliv.) Diels), Zhi mu (Anemarrhenae Rhizoma, *Anemarrhena asphodeloides* Bge.), Huang bai (Phellodendri Chinensis Cortex, *Phellodendron chinense* Schneid.) |
| Zhou & Li 2016 [18] | Decoction | Chinese herbal formula | Chai hu (Bupleuri Radix, *Bupleurum chinense* DC. or *Bupleurum scorzonerifolium* Willd.), Bai shao (Paeoniae Radix Alba, *Paeonia lactiflora* Pall.), Shu di huang (Rehmanniae Radix Praeparata, *Rehmannia glutinosa* Libosch.), Yin yang huo (Epimedii Folium, *Epimedium brevicornum* Maxim., *Epimedium sagittatum* (Sieb. et Zucc.) Maxim., *Epimedium pubescens* Maxim. or *Epimedium koreanum* Nakai), Yi mu cao (Leonuri herba, *Leonurus japonicus* Houtt.), Dang gui (Angelicae Sinensis Radix, *Angelica sinensis* (Oliv.) Diels), Nü zhen zi (Ligustri Lucidi Fructus, *Ligustrum lucidum* Ait.), Da zao (Jujubae Fructus, *Ziziphus jujuba* Mill.), Xiang fu (Cyperi Rhizoma, *Cyperus rotundus* L.), Tu si zi (Cuscutae Semen, *Cuscuta australis* R. Br. or *Cuscuta chinensis* Lam.), Gan cao (Glycyrrhizae Radix et Rhizoma, *Glycyrrhiza uralensis* Fisch., *Glycyrrhiza inflata* Bat. or *Glycyrrhiza glabra* L.), Dang shen (Codonopsis Radix, *Codonopsis pilosula* (Franch.) Nannf., *Codonopsis pilosula* Nannf. var. modesta (Nannf.) L. T. Shen or *Codonopsis tangshen* Oliv.), Hong hua (Carthami Flos, *Carthamus tinctorius* L.), Dan shen (Salviae Miltiorrhizae Radix et Rhizoma or *Salvia miltiorrhiza* Bge.) |
| Zhou et al. 2007 [19] | Granule | Geng Nian An granule | Tu si zi (Cuscutae Semen, *Cuscuta australis* R. Br. or *Cuscuta chinensis* Lam.), Fu pen zi (Rubi Fructus, *Rubus chingii* Hu), Dang gui (Angelicae Sinensis Radix, *Angelica sinensis* (Oliv.) Diels), Bai shao (Paeoniae Radix Alba, *Paeonia lactiflora* Pall.), Chai hu (Bupleuri Radix, *Bupleurum chinense* DC. or *Bupleurum scorzonerifolium* Willd.), Nü zhen zi (Ligustri Lucidi Fructus, *Ligustrum lucidum* Ait.), Shan yao (Dioscoreae Rhizoma, *Dioscorea opposita* Thunb.), Gui zhi (Cinnamomi Ramulus, *Cinnamomum cassia* Presl), Mu li (Ostreae Concha, *Ostrea gigas* Thunberg, *Ostrea talienwhanensis* Crosse, *Ostrea rivularis* Gould), Dang shen (Codonopsis Radix, *Codonopsis pilosula* (Franch.) Nannf., *Codonopsis pilosula* Nannf. var. modesta (Nannf.) L. T. Shen or *Codonopsis tangshen* Oliv.) |

# References

1. Azizi H, Feng Liu Y, Du L, Hua Wang C, Bahrami-Taghanaki H, Ollah Esmaily H, et al. Menopause-related symptoms: Traditional Chinese medicine vs hormone therapy. Alternative therapies in health and medicine. 2011;17(4): 48-53. PMID: 22314633.

2. Chen HL. 50 Cases with menopausal syndrome treated by Kun Tai capsule. China Pharmaceuticals. 2014;23(14): 108-9.

3. Davis SR, Briganti EM, Chen RQ, Dalais FS, Bailey M, Burger HG. The effects of Chinese medicinal herbs on postmenopausal vasometer symptoms of Australian women: a randomised controlled trial. Medical Journal of Australia. 2001;174(2): 68-71. PMID: 0021597.

4. Fu LH, Fan LY, Qiao QZ, Zhang YX, Feng LX. Observation of clinical efficacy on perimenopausal syndrome treated with herbal formulas. World Journal of Integrated Traditional and Western Medicine. 2015;(1): 89-91.

5. Fu SF, Zhao YQ, Ren M, Zhang JH, Wang YF, Han LF, et al. A randomized, double-blind, placebo-controlled trial of Chinese herbal medicine granules for the treatment of menopausal symptoms by stages. Menopause. 2016;23(3): 311-23. doi: 10.1097/GME.0000000000000534. PMID: 114026755.

6. Grady D, Sawaya GF, Johnson KC, Koltun W, Hess R, Vittinghoff E, et al. MF101, a selective estrogen receptor beta modulator for the treatment of menopausal hot flushes: A phase II clinical trial. Menopause. 2009;16(3): 458-65. doi: 10.1097/gme.0b013e31818e64dd. PMID: CN-00703125.

7. Haines CJ, Lam PM, Chung TK, Cheng KF, Leung PC. A randomized, double-blind, placebo-controlled study of the effect of a Chinese herbal medicine preparation (Dang Gui Buxue Tang) on menopausal symptoms in Hong Kong Chinese women. Climacteric. 2008;11(3): 244-51. doi: 10.1080/13697130802073029. PMID: 18568789.

8. Li HY, Qiu WF, Xu YP, Zhao DN, He J, Ning XP. Therapeutic effects of Dingkundan on female climacteric symptoms. Hebei Medical Journal. 2018;40(23): 3610-2.

9. Liu XY. Clinical observation of modified Danzhi Xiaoyao San in the treatment of climacteric syndrome in 64 participants. Guiding Journal of Traditional Chinese Medicine and Pharmacy. 2008;14(9): 47-8.

10. Luan YQ, Yang X, Peng XL, Fu C, Meng ZX, Zhang J. Double-blind double dummy randomized parallel clinical trial of Kun-Tai capsule in the treatment of perimenopausal symptoms. Chinese Journal of Clinical Pharmacology. 2004;20(6): 452-5.

11. Nedeljkovic M, Tian L, Ji P, Deglon-Fischer A, Stute P, Ocon E, et al. Effects of acupuncture and Chinese herbal medicine (Zhi Mu 14) on hot flushes and quality of life in postmenopausal women: Results of a four-arm randomized controlled pilot trial. Menopause. 2014;21(1): 15-24. doi: 10.1097/GME.0b013e31829374e8. PMID: 23676632.

12. Plotnikoff GA, Watanabe K, Torkelson C, La Valleur J, Radosevich DM, Plotnikoff GA, et al. The TU-025 keishibukuryogan clinical trial for hot flash management in postmenopausal women: Results and lessons for future research. Menopause. 2011;18(8): 886-92. doi: 10.1097/gme.0b013e31821643d9. PMID: 108191282.

13. Sun AJ, Wang YP, Gu B, Zheng TP, Lin SQ, Bai WP, et al. A multi-center, randomized, controlled and open clinical trial of Heyan Kuntai Capsule and hormone therapy in perimenopausal women. Chinese Journal of Integrative Medicine. 2018;24(7): 487-93.

14. Ushiroyama T, Ikeda A, Sakuma K, Ueki M. Comparing the effects of estrogen and an herbal medicine on peripheral blood flow in post-menopausal women with hot flashes: Hormone replacement therapy and Gui-zhi-fu-ling-wan, a kampo medicine. American Journal of Chinese Medicine. 2005;33(2): 259-67. doi: 10.1142/S0192415X05002813.

15. van der Sluijs CP, Bensoussan A, Chang S, Baber R. A randomized placebo-controlled trial on the effectiveness of an herbal formula to alleviate menopausal vasomotor symptoms. Menopause. 2009;16(2): 336-44. doi: 10.1097/gme.0b013e3181883dc1.

16. Xia Y, Zhao Y, Ren M, Zhang J, Wang Y, Chang Y, et al. A randomized double-blind placebo-controlled trial of a Chinese herbal medicine preparation (Jiawei Qing'e Fang) for hot flashes and quality of life in perimenopausal women. Menopause. 2012;19(2): 234-44. doi: 10.1097/gme.0b013e3182273177. PMID: 22089177.

17. Zhong LL, Tong Y, Tang GW, Zhang ZJ, Choi WK, Cheng KL, et al. A randomized, double-blind, controlled trial of a Chinese herbal formula (Er-Xian decoction) for menopausal symptoms in Hong Kong perimenopausal women. Menopause. 2013;20(7): 767-76. doi: 10.1097/GME.0b013e31827cd3dd. PMID: 23793167.

18. Zhou YJ, Li J. Combination of ear pallet and Chinese herbal medicine in treating climacteric syndrome. Jiangxi Medical Journal. 2016;51(2): 170-2.

19. Zhou J, Qu F, Nan R, Tang D. The effect of chinese medicinal herbs in relieving menopausal symptoms in ovariectomized chinese women. Explore. 2007;3(5): 478-84. doi: 10.1016/j.explore.2007.06.002. PMID: 17905357.
