# Supplementary material for: Chinese herbal formulae for the treatment of menopausal hot flushes: A systematic review and meta-analysis
Source: PLoS One. 2019 Sep 19;14(9):e0222383. doi: 10.1371/journal.pone.0222383 (PMC6752783; doi:10.1371/journal.pone.0222383)
Supplement: S3 Table — (DOCX) [file pone.0222383.s004.docx]

Supplementary material

## S3 Table. Details of adverse events reported in the included studies

| Study ID | Treatment group | Control group |
| --- | --- | --- |
| Azizi et al. 2011 [1] | No adverse events observed | No adverse events observed |
| Chen 2014 [2] | Not reported | Not reported |
| Davis et al. 2001 [3] | Abdominal bloating (n=1), lower abdominal pain and loose stool (n=2), headache, joint pain or dizziness (n=6) | Abdominal bloating (n=3), headache, joint pain or dizziness (n=9) |
| Fu et al 2015a [4] | No adverse events observed | No adverse events observed |
| Fu et al. 2015b [5] | Palpitations (n=5), nausea/discomfort in stomach (n=5), dry mouth/dental ulcers/swollen gums (n=17), constipation (n=5), rashes (n=4), hypersomnia (n=4), and insomnia (n=3), higher transaminase levels (n=16, intervention groups were not specified) | Nausea/discomfort in stomach (n=1), dry mouth/dental ulcers/swollen gums (n=10), constipation (n=1), hypersomnia (n=1), insomnia (n=1) |
| Grady et al. 2009 [6] | Idiopathic pancreatitis (n=1); axillary adenocarcinoma of the skin or subcutaneous tissue (n=1), loose stool (n=17) | Cellulitis (n=1), loss stool (n=2) |
| Haines et al. 2008 [7] | Rectal bleeding (n=1), constipation (n=1), epigastric discomfort (n=1), hypercholesterolemia (n=1), per rectum bleeding (n=1) | Elevation of hepatic enzymes (n=1), epigastria pain (n=2), insomnia (n=2), stomach upset (n=1), skin tags (n=1), influenza (n=1), pituitary adenoma (n=1), hepatic enzyme increase (n=1) |
| Li et al. 2018 [8] | No adverse events observed | Vaginal bleeding (n=2), breast tenderness (n=3) |
| Liu 2008 [9] | Nausea (n=1) | Headache (n=1), nausea (n=3), vaginal bleeding (n=2) |
| Luan et al. 2004 [10] | Stomach discomfort (n=2), vaginal bleeding (n=4), breast tenderness (n=5) | Stomach discomfort (n=2), vaginal bleeding (n=6), breast tenderness (n=7) |
| Nedeljkovic et al. 2013 [11] | Dysplasia of the squamous epithelium (n=1) | No adverse events observed |
| Plotnikoff et al. 2011 [12] | Diarrhea (n=25) | Diarrhea (n=1) |
| Sun et al. 2018 [13] | Stomach discomfort (n=5), breast distending pain (n=26) | Stomach discomfort (n=1), breast distending pain (n=59) |
| Ushiroyama et al. 2005 [14] | Not reported | Not reported |
| van der Sluijs et al. 2009 [15] | 57% participants (n=26) reported adverse events: headaches and gastrointestinal complaints (mild to moderate in severity) | 48% participants (n=22)  reported adverse events: headaches and gastrointestinal complaints (mild to moderate in severity) |
| Xia et al. 2012 [16] | Rash (n=1), mild hydrosalpinx (n=1), elevated ALT (n=1) | Herpes zoster (n=1), abnormal vaginal bleeding (n=1), elevated AST (n=1) |
| Zhong et al. 2013 [17] | Mild diarrhea (n=1), headache (n=2), worsening of depression (n=2), stomach ache (n=1) | Headache (n=1), worsening of insomnia (n=2), worsening of hot flushes (n=3) |
| Zhou & Li 2016 [18] | No adverse events observed | No adverse events observed |
| Zhou et al. 2007 [19] | No adverse events observed | No adverse events observed |

# References

1. Azizi H, Feng Liu Y, Du L, Hua Wang C, Bahrami-Taghanaki H, Ollah Esmaily H, et al. Menopause-related symptoms: Traditional Chinese medicine vs hormone therapy. Alternative therapies in health and medicine. 2011;17(4): 48-53. PMID: 22314633.

2. Chen HL. 50 Cases with menopausal syndrome treated by Kun Tai capsule. China Pharmaceuticals. 2014;23(14): 108-9.

3. Davis SR, Briganti EM, Chen RQ, Dalais FS, Bailey M, Burger HG. The effects of Chinese medicinal herbs on postmenopausal vasometer symptoms of Australian women: a randomised controlled trial. Medical Journal of Australia. 2001;174(2): 68-71. PMID: 0021597.

4. Fu LH, Fan LY, Qiao QZ, Zhang YX, Feng LX. Observation of clinical efficacy on perimenopausal syndrome treated with herbal formulas. World Journal of Integrated Traditional and Western Medicine. 2015;(1): 89-91.

5. Fu SF, Zhao YQ, Ren M, Zhang JH, Wang YF, Han LF, et al. A randomized, double-blind, placebo-controlled trial of Chinese herbal medicine granules for the treatment of menopausal symptoms by stages. Menopause. 2016;23(3): 311-23. doi: 10.1097/GME.0000000000000534. PMID: 114026755.

6. Grady D, Sawaya GF, Johnson KC, Koltun W, Hess R, Vittinghoff E, et al. MF101, a selective estrogen receptor beta modulator for the treatment of menopausal hot flushes: A phase II clinical trial. Menopause. 2009;16(3): 458-65. doi: 10.1097/gme.0b013e31818e64dd. PMID: CN-00703125.

7. Haines CJ, Lam PM, Chung TK, Cheng KF, Leung PC. A randomized, double-blind, placebo-controlled study of the effect of a Chinese herbal medicine preparation (Dang Gui Buxue Tang) on menopausal symptoms in Hong Kong Chinese women. Climacteric. 2008;11(3): 244-51. doi: 10.1080/13697130802073029. PMID: 18568789.

8. Li HY, Qiu WF, Xu YP, Zhao DN, He J, Ning XP. Therapeutic effects of Dingkundan on female climacteric symptoms. Hebei Medical Journal. 2018;40(23): 3610-2.

9. Liu XY. Clinical observation of modified Danzhi Xiaoyao San in the treatment of climacteric syndrome in 64 participants. Guiding Journal of Traditional Chinese Medicine and Pharmacy. 2008;14(9): 47-8.

10. Luan YQ, Yang X, Peng XL, Fu C, Meng ZX, Zhang J. Double-blind double dummy randomized parallel clinical trial of Kun-Tai capsule in the treatment of perimenopausal symptoms. Chinese Journal of Clinical Pharmacology. 2004;20(6): 452-5.

11. Nedeljkovic M, Tian L, Ji P, Deglon-Fischer A, Stute P, Ocon E, et al. Effects of acupuncture and Chinese herbal medicine (Zhi Mu 14) on hot flushes and quality of life in postmenopausal women: Results of a four-arm randomized controlled pilot trial. Menopause. 2014;21(1): 15-24. doi: 10.1097/GME.0b013e31829374e8. PMID: 23676632.

12. Plotnikoff GA, Watanabe K, Torkelson C, La Valleur J, Radosevich DM, Plotnikoff GA, et al. The TU-025 keishibukuryogan clinical trial for hot flash management in postmenopausal women: Results and lessons for future research. Menopause. 2011;18(8): 886-92. doi: 10.1097/gme.0b013e31821643d9. PMID: 108191282.

13. Sun AJ, Wang YP, Gu B, Zheng TP, Lin SQ, Bai WP, et al. A multi-center, randomized, controlled and open clinical trial of Heyan Kuntai Capsule and hormone therapy in perimenopausal women. Chinese Journal of Integrative Medicine. 2018;24(7): 487-93.

14. Ushiroyama T, Ikeda A, Sakuma K, Ueki M. Comparing the effects of estrogen and an herbal medicine on peripheral blood flow in post-menopausal women with hot flashes: Hormone replacement therapy and Gui-zhi-fu-ling-wan, a kampo medicine. American Journal of Chinese Medicine. 2005;33(2): 259-67. doi: 10.1142/S0192415X05002813.

15. van der Sluijs CP, Bensoussan A, Chang S, Baber R. A randomized placebo-controlled trial on the effectiveness of an herbal formula to alleviate menopausal vasomotor symptoms. Menopause. 2009;16(2): 336-44. doi: 10.1097/gme.0b013e3181883dc1.

16. Xia Y, Zhao Y, Ren M, Zhang J, Wang Y, Chang Y, et al. A randomized double-blind placebo-controlled trial of a Chinese herbal medicine preparation (Jiawei Qing'e Fang) for hot flashes and quality of life in perimenopausal women. Menopause. 2012;19(2): 234-44. doi: 10.1097/gme.0b013e3182273177. PMID: 22089177.

17. Zhong LL, Tong Y, Tang GW, Zhang ZJ, Choi WK, Cheng KL, et al. A randomized, double-blind, controlled trial of a Chinese herbal formula (Er-Xian decoction) for menopausal symptoms in Hong Kong perimenopausal women. Menopause. 2013;20(7): 767-76. doi: 10.1097/GME.0b013e31827cd3dd. PMID: 23793167.

18. Zhou YJ, Li J. Combination of ear pallet and Chinese herbal medicine in treating climacteric syndrome. Jiangxi Medical Journal. 2016;51(2): 170-2.

19. Zhou J, Qu F, Nan R, Tang D. The effect of chinese medicinal herbs in relieving menopausal symptoms in ovariectomized chinese women. Explore. 2007;3(5): 478-84. doi: 10.1016/j.explore.2007.06.002. PMID: 17905357.
